# The effects of different carbon-fiber plate shapes in shoes on lower limb biomechanics following running-induced fatigue

**DOI:** 10.3389/fbioe.2025.1539976

**Published:** 2025-02-11

**Authors:** Yufan Xu, Chengyuan Zhu, Yufei Fang, Zhenghui Lu, Yang Song, Chen Hu, Dong Sun, Yaodong Gu

**Affiliations:** ^1^ Faculty of Sports Science, Ningbo University, Ningbo, China; ^2^ Department of Radiology, Ningbo No. 2 Hospital, Ningbo, China; ^3^ Faculty of Engineering, University of Pannonia, Veszprem, Hungary; ^4^ Department of Material Science and Technology, Audi Hungaria Faculty of Automotive Engineering, Széchenyi István University, Győr, Hungary; ^5^ Department of Biomedical Engineering, Faculty of Engineering, The Hong Kong Polytechnic University, Kowloon, Hong Kong SAR, China

**Keywords:** lower limb biomechanics, carbon-fiber plate (CFP), running-induced fatigue, running performance, injury

## Abstract

Different shapes of carbon-fiber plates (CFPs) are likely to affect lower limb biomechanics, particularly under conditions of running-induced fatigue, and potentially impact runners’ performance and risk of injury. However, no studies have yet elucidated the precise effects of CFP shapes on the lower limb biomechanical characteristics subsequent to running-induced fatigue. The purpose of this study was to investigate the effects of different CFP shapes in running shoes on the lower limb biomechanics of runners following running-induced fatigue. 12 male runners (aged 21.8 ± 1.3 years, mass 59.1 ± 4.1 kg, height 168.9 ± 2.2 cm, weekly running distance 68.8 ± 5.5 km/week) were recruited for this study. Two-way repeated measures ANOVA was used to compare kinematic and kinetic data, while SPM (Statistical Parametric Mapping) was used to assess the activation levels of lower limb muscles. Compared to wearing flat CFP shoes (“Flat”), wearing curved CFP shoes (“Curve”) resulted in a significant reduction in the hip (p = 0.034) and knee contact angle (p < 0.000), as well as a significant decrease in the hip flexion moment (p = 0.008). The activation level of the tibialis anterior (TA) was significantly higher when wearing “Curve” in pre-fatigue compared to “Flat”, whereas the opposite was observed post-fatigue. The curved CFP altered the bending angle of the forefoot, thereby significantly reducing the joint angles and joint moments of the hip and knee.

## 1 Introduction

The popularization and specialization of running led runners to focus more on running shoes, which directly contacted the ground as essential equipment (Bermon, 2021; Lin et al., 2022; Song et al., 2022). Carbon-Fiber Plate Shoes (CPS), designed with lightweight, elastic, and highly cushioned foam midsoles, breathable upper materials, and a rigid carbon-fiber plate (CFP) insert, have been shown to reduce energy consumption during running and enhance running economy (RE) (Worobets et al., 2014; Onodera et al., 2017; Ortega et al., 2021; Subramanium et al., 2021; Gao et al., 2024). The Nike Vaporfly 4% demonstrated a 4% improvement in RE (Hoogkamer et al., 2018), and marathon world record-holder Eliud Kipchoge wore this shoe when he set the record at 2:01:39. This groundbreaking discovery represents a milestone in the development of CPS, driving its increasing popularity in marathon running.

Various variables related to CPS, such as running shoes cushioning (Borgia et al., 2020), stiffness (Rodrigo-Carranza et al., 2022), heel-to-toe drop (Malisoux et al., 2016), and shoes aging (Song et al., 2025; Chambon et al., 2014), have been investigated, but greater emphasis has been placed on the design of the CFP, which has been tailored by designers to meet the individualized requirements of runners for training and competition (Stefanyshyn and Nigg, 2000; Flores et al., 2021; Fu et al., 2021). These design variations include altering plate stiffness (Roy and Stefanyshyn, 2006; Madden et al., 2016), adjusting the plate’s position within the midsole (below, between, or above the midsole) (Stefanyshyn and Nigg, 2000; Madden et al., 2016; Oh and Park, 2017), and modifying the plate’s shape (curved or flat) (Ruiz-Alias et al., 2023). During the stance phase of running, the CFP plays a critical role in influencing the movement of the metatarsophalangeal joint (MTP) (Cigoja et al., 2020; Deschamps et al., 2020; Hoitz et al., 2020; Sun et al., 2020). Compared to lower positions, a higher plate position is associated with a significant reduction in lower limb joint moments and positive knee work, thereby rendering it a preferable configuration for improving running performance (Flores et al., 2021). In terms of plate shape, a review indicated that footwear with a curved CFP improved running economy by 3.45%, while flat CFP footwear provided only a slight improvement of 0.19% (Rodrigo-Carranza et al., 2022). Additionally, research by Song et al. (2024) showed that, compared to flat CFP, curved CFP further reduced forefoot loading during running. These findings demonstrate that CFP design has a significant impact on lower limb biomechanics, with even minor design differences leading to notable variations in effects.

Previous studies have demonstrated that although CPS, particularly those with curved designs, can improve RE to a certain degree, their design may contribute to the acceleration of lower limb fatigue (Agresta et al., 2022; Hata et al., 2022). Fatigue was found to reduce ground contact time and peak knee flexion angle during the stance phase (Morin et al., 2011; Chan-Roper et al., 2012), impair the muscles’ ability to maintain joint stability (Warden et al., 2014; Apte et al., 2021), and lower the symmetry of lower limbs in running gait (Gao et al., 2022). CPS were also shown to cause injuries such as navicular stress fractures and plantar fasciitis in individuals with limited long-distance running experience or those unaccustomed to CFP footwear (McKenzie et al., 1985; Kiuru et al., 2004; Tenforde et al., 2023). Research on the potential performance enhancements provided by CPS and the risks of lower limb injuries linked to running-induced fatigue is continuously advancing. However, studies investigating the effects of varying CFP shapes in running shoes and running-induced fatigue on lower limb biomechanical characteristics have typically treated these factors independently. No study has yet examined how the combined effects of CFP shape and running-induced fatigue in shoes with embedded carbon plates impact lower limb biomechanical characteristics, running performance, and the risk of running-related injuries. Therefore, the purpose of this study was to investigate the effects of different CFP shapes in running shoes on the lower limb biomechanics of runners following running-induced fatigue. Based on previous studies, it was hypothesized that variations in CFP shapes would influence the distance between the cushioning foam material and the ground, resulting in reduced ground impact at the MTP during initial contact for runners wearing curved CPS as opposed to flat CPS. Furthermore, irrespective of fatigue, curved CPS exhibited superior performance compared to flat CPS. We speculated that the curved CPS would facilitate better leverage at the MTP, thereby increasing its range of motion (ROM) and positive work. Additionally, to cope with varying impacts and stabilize the lower limb joints, the activation levels of the calf muscles would exhibit differences.

This study will compare the differences in lower limb kinematics, dynamics, and muscle activation characteristics before and after running-induced fatigue, using running shoes with different CFP shapes. The impact of CFP shape on lower limb fatigue patterns will be assessed, providing data to support improvements in running performance and injury prevention. The study will reveal the influence of CFP and fatigue status on performance and injury risk, offering scientific evidence for the design of sports footwear and assisting designers in developing running shoes that are more aligned with biomechanical principles.

## 2 Materials and methods

### 2.1 Participants

An effect size of 0.78 was calculated from the results of the preliminary experiment. To enhance the robustness of the experimental results, the effect size was adjusted to 0.70. According to the result from G-power 3.1 (x64, Heinrich Heine University Düsseldorf, Germany), the minimum sample size required for this study was determined to be 10 participants (with (1-β) = 0.95, significance level α = 0.05, two groups, and two measurements). Therefore, this study recruited 12 male young runners at the elite level or above as participants. Therefore, we recruited 12 male mass elite runners (aged 21.8 ± 1.3 years, mass 59.1 ± 4.1 kg, height 168.9 ± 2.2 cm, weekly running distance 68.8 ± 5.5 km/week, personal best of half marathon 78.75 ± 2.27 min, Personal best of half marathon full marathon 175.28 ± 3.51 min, as participants through personal contacts and Ningbo Marathon Association. Among the participants, 10 habitually employ a forefoot strike, while two employ a rearfoot strike. All runners selected to participate in this experiment meet the following criteria: fitting 41 EU size shoes, the dominant leg is right leg, the best personal record for the half marathon is sub-85 min (or equivalent) and/or for the full marathon is sub-180 min (or equivalent) based on the 2024 classification standards for mass participants issued by the Chinese Athletics Association (RunChina, 2024), and free from any injuries sustained in the lower limbs in the past 6 months. Written informed consent was provided by all participants for the experimental procedures, which were approved by the Ningbo University Ethics Committee (TY2024210). This study was compiled with the Declaration of Helsinki.

### 2.2 Shoe conditions

**FIGURE 1:**
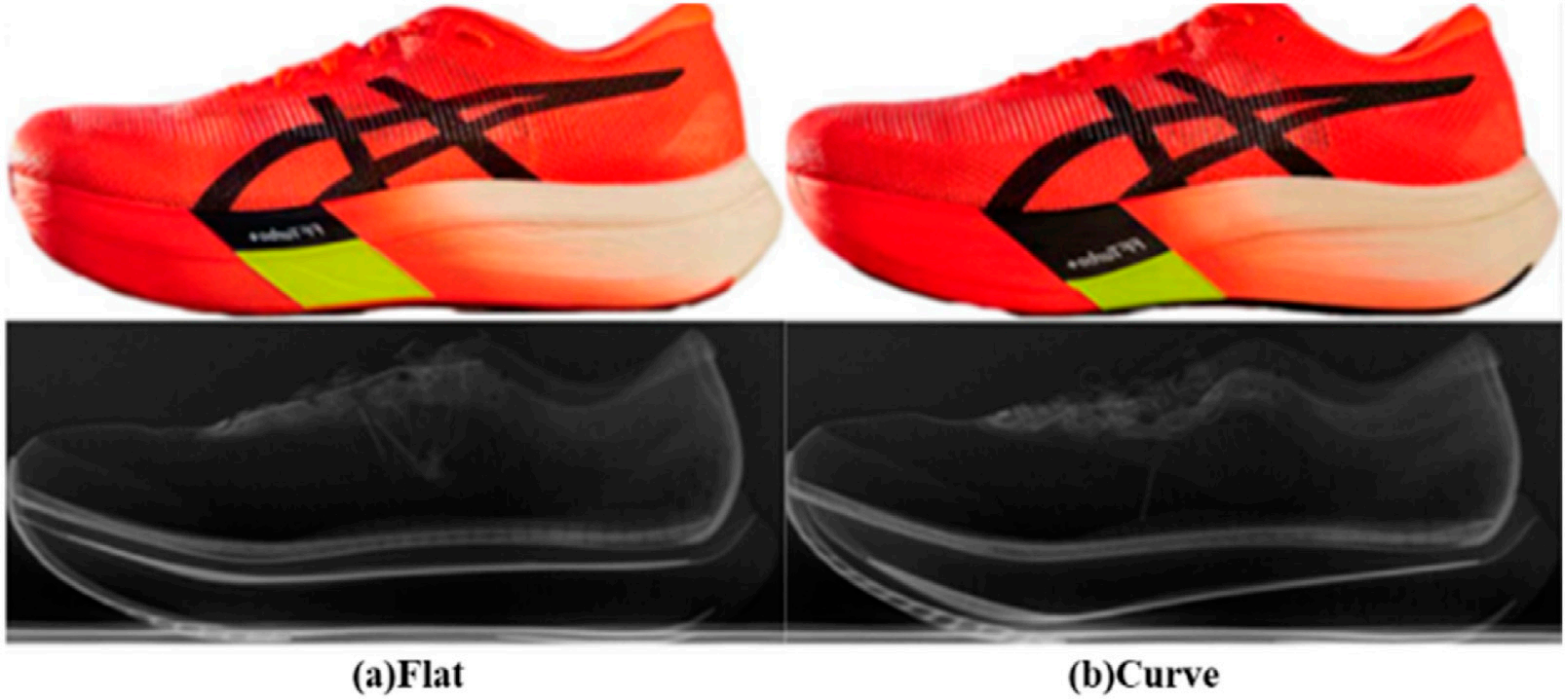
Image of the shoes prototype utilized in the experiment. The upper shows the exterior view of the shoes, while the lower displays the internal CFP captured. **(A)** Is the “Flat”, and **(B)** is the “Curve”.

Two types of running shoes were employed in this study: ASICS METASPEED SKY PARIS and ASICS METASPEED EDGE PARIS. Both experimental shoe models exhibit a similar appearance, featuring FF (Flyte Foam) Turbo Plus as the midsole material and a rigid embedded CFP. The difference is that the former weighs 183 g/shoe and has a flatter CFP, hence abbreviated as “Flat”, while the latter weighs 185 g/shoe and features a more curved carbon plate at the forefoot, hence abbreviated as “Curve”. To eliminate differences in shoe weight, a 2 g muscle-effect patch was applied to the “Flat” to balance the mass. In addition, manual measurements revealed that the “Curve” exhibits a toe box 3 mm higher than that of the “Flat.” The total running distance for any pair of shoes was limited to no more than 50 km (Figure 1).

### 2.3 Experimental set-up and protocol

**FIGURE 2:**
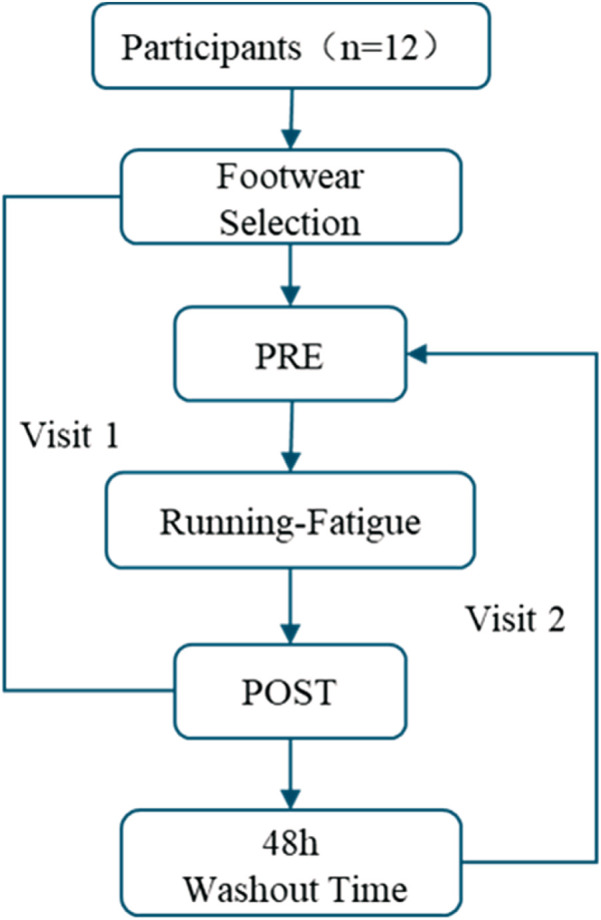
The flowchart of experiment.

Each participant was required to complete two laboratory visits as part of this study. The procedures were common on both visits, with the only difference being that participants randomly wore one pair of shoes on Visit1 and wore the other pair on Visit2. The randomization process primarily involved ensuring that participants were unaware of the specific differences between the two pairs of experimental shoes both before and during the experiment. The order of shoe wear for the two experimental sessions was determined according to the participants’ subjective preferences, thereby achieving randomization in this study. The flowchart of experiment as shown in Figure 2.

**FIGURE 3:**
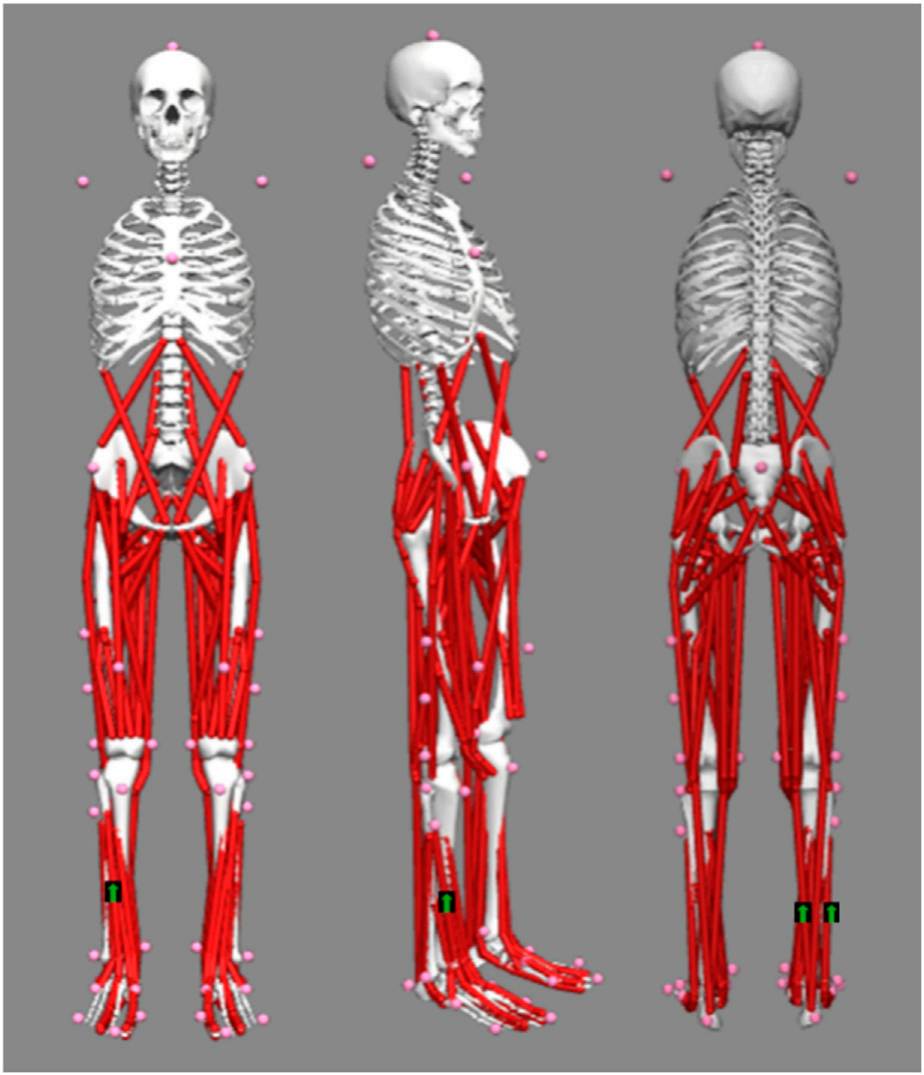
Illustration of reflective markers and electrodes placement locations.

Participants performed a 10-min running warm-up at a self-selected speed in their own shoes on a treadmill (Saturn 300/100 r, h/p/cosmos, Germany). Following a 5-min cool-down, the anatomical positions of the right tibialis anterior (TA), medial gastrocnemius (MG), and lateral gastrocnemius (LG) were identified for electromyography (EMG) electrode placement (Delsys, Natick, MA, United States). Before the placement of the electrodes, the hair was wiped off with a razor and cleaned with an alcohol wipe. To facilitate motion capture, a total of 38 reflective markers were attached to anatomical landmarks according to the 2,392 model (Delp et al., 1990). The placement locations of reflective markers and EMG electrodes are illustrated in Figure 3.

The fatigue intervention began with a treadmill set to a 1° incline to simulate an outdoor running environment (Fourchet et al., 2015). Participants began walking on a treadmill at a speed of 6 km/h, with the speed increasing by 1 km/h every 2 min, enabling them to transition into running. Heart rate (HR) data were collected using Polar heart rate monitors (Polar Electro, Kempele, Finland), and RPE (Rate of Perceived Exertion) scores were responded by the participants every minute throughout the intervention. The RPE score is a rating method used to measure an individual’s subjective perception of effort during exercise or physical activity, ranging from 6 (almost no effort) to 20 (maximum effort). The treadmill speed stabilized when participants’ RPE scores reached 13 (indicating slight fatigue). Participants then continued running at this steady speed until their heart rate (HR) reached 85% of their maximum heart rate (calculated as 220 minus age) and maintained this level for 2 min without significant decline, completing the fatigue protocol (Hajiloo et al., 2020). Markers and EMG electrodes often dislodged during the fatigue intervention due to sweating and movement, therefore, to reduce the preparation time after the intervention, a black marker pen was used to mark the center locations of the markers on the skin before the formal experiment.

Data collection began with a static calibration trial, which was subsequently used to scale a musculoskeletal model. The participants along an 18-m track at a self-selected speed. A 3D motion capture system with 10 cameras (Vicon Metrics Ltd., Oxford, United Kingdom) and 2 force plates (Kistler, Winterthur, Switzerland) recorded marker trajectories and ground reaction forces (GRF) during running at sampling frequencies of 200 Hz and 2000 Hz, respectively and Delsys recorded EMG signals synchronously at a sampling frequency of 2000 Hz. A successful test was defined as the dominant leg fully landed on the force plate.

Once the pre-fatigue data collection was completed, the fatigue intervention was carried out. After the fatigue intervention, any dropped markers and EMG electrodes were replaced, and the data collection process was repeated following the same procedures as pre-fatigue. To minimize measurement errors and enhance data accuracy, three successful data sets were collected during each test. Following the completion of Visit 1, a 48-h washout period was implemented before the commencement of Visit 2 (Ruiz-Alias et al., 2024).

### 2.4 Data analysis

The kinematic data of right hip, knee, ankle, and MTP during the stance phase, as well as GRF, were collected as participants passed through the force plates. Vicon Nexus (2.15.0 x64, Vicon Motion Systems, Oxford, United Kingdom) was used to preprocess the kinematic data, with a vertical GRF threshold of 10N set for stance phase detection, resulting in c3d. files. The custom code in MATLAB (R2022a, The MathWorks Inc., Natick, Middlesex, MA, United States) was used to convert the files. The angle and the moments of the hip, the knee, the ankle and the MTP joints of the right lower limb were calculated in the sagittal plane during the stance phase using the inverse kinematics and inverse dynamics algorithm tools in OpenSim 4.3. Custom Python code was employed to filter the kinematic data at 6 Hz, while the GRF data were low-pass filtered at 50 Hz with a critically damped filter. In the presentation of lower limb biomechanical parameters such as joint angles and joint moments, positive values represent hip flexion, knee extension, and dorsiflexion of the ankle and MTP, while negative values represent hip extension, knee flexion, and plantarflexion of the ankle and MTP. Based on previous research, the vertical average loading rate (VALR) was considered as a representative value of the loading rate (LR) of the GRF (Willson et al., 2014). The VALR is the slope of the line connecting the 20% point and the 80% point of the 13% stance phase (An et al., 2015). Joint power is determined using the following equation:

P=M⋅ω

**P** represents the joint power (units: W/kg), **M** denotes the joint moment (units: Nm), **ω** denotes the joint angle velocity (units: rad/s).

The work performed by the joint is determined using the following equation:

$$W=\int_{t1}^{t2}P\;dt$$

**W** represents the work performed by the joint (units: J/kg), **P** denotes the joint power, **t1** and **t2** denote the start and end times of the integration interval.

EMG data were filtered using a band-pass filter between 20 and 450 Hz. To perform the linear envelope process, the EMG data were full-wave rectified and subsequently low-pass filtered at a cutoff frequency of 20 Hz using a fourth-order Butterworth filter. The submaximal method was used for normalization, where the maximum EMG signal for each muscle was first calculated under each of the conditions in our study, and each data point was then divided by the maximum value for the corresponding condition to obtain the muscle activation as a percentage of the maximum muscle activity for that condition (Hajiloo et al., 2020). The collected EMG signals were time-normalized and divided into 101 points.

### 2.5 Statistical analysis

The statistical analyses were performed by SPSS (26, IBM Corp., Armonk, NY, United States), and the obtained parameters are expressed as mean ± standard deviation (X±SD), the Shapiro-Wilk test was employed. A two-way repeated measures ANOVA was used to evaluate the main effects of “CFP shape” and “fatigue,” as well as the interaction between these factors on biomechanical variables. Alpha levels were set at 0.05. When the interaction effect was significant, *post hoc* pairwise comparisons were conducted, and the alpha level was adjusted to <0.0125 using the Bonferroni correction.

A Statistical Parametric Mapping (SPM) procedure (Pataky et al., 2013) was used to assess the main effects of “CFP shape” and “fatigue”, as well as their interaction, on the EMG signals of the TA and gastrocnemius muscles during the stance phase of running. SPM tests were calculated using SPM1d v0.4 for MATLAB (Pataky et al., 2015). The significance level for all statistical tests was set at 0.05.

## 3 Results

### 3.1 Interaction effect

**FIGURE 4:**
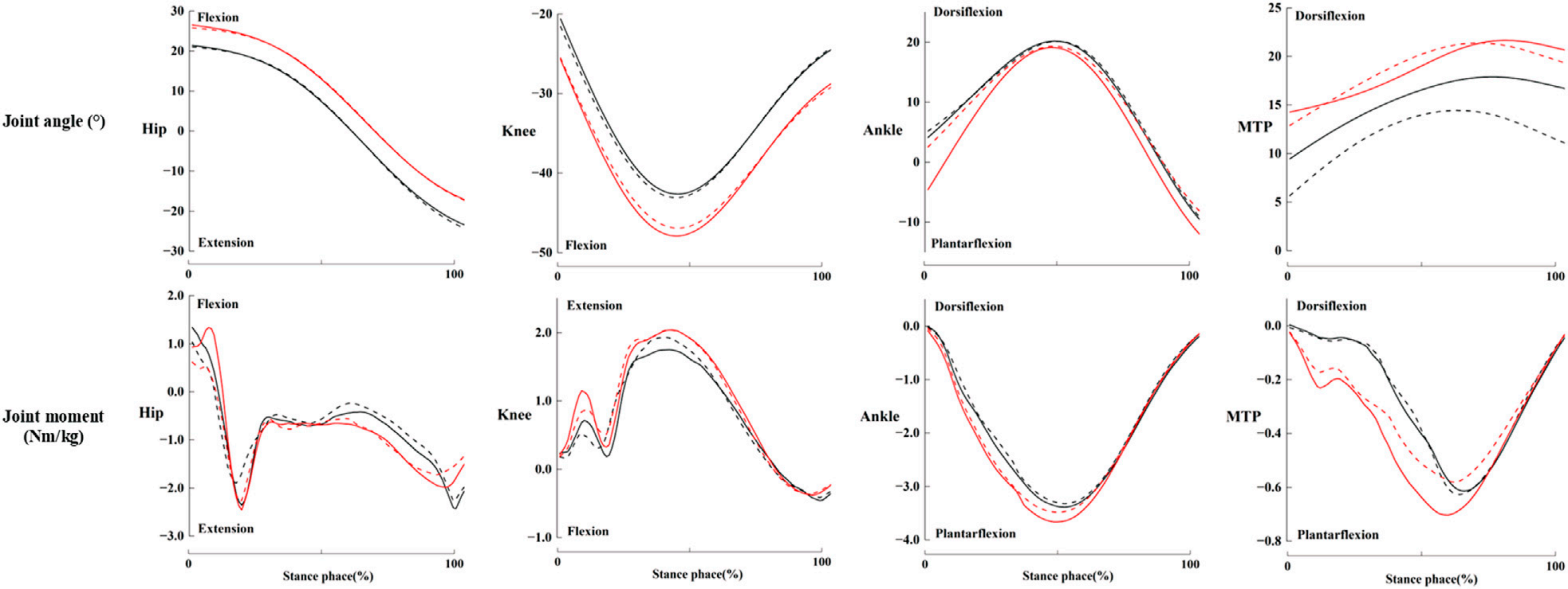
Joint angle (top column) and joint moment (bottom column) for the hip, knee, ankle, and MTP. Red indicates wearing “Flat”, black indicates wearing “Curve”; solid lines represent pre-fatigue, and dashed lines represent post-fatigue.

**FIGURE 5:**
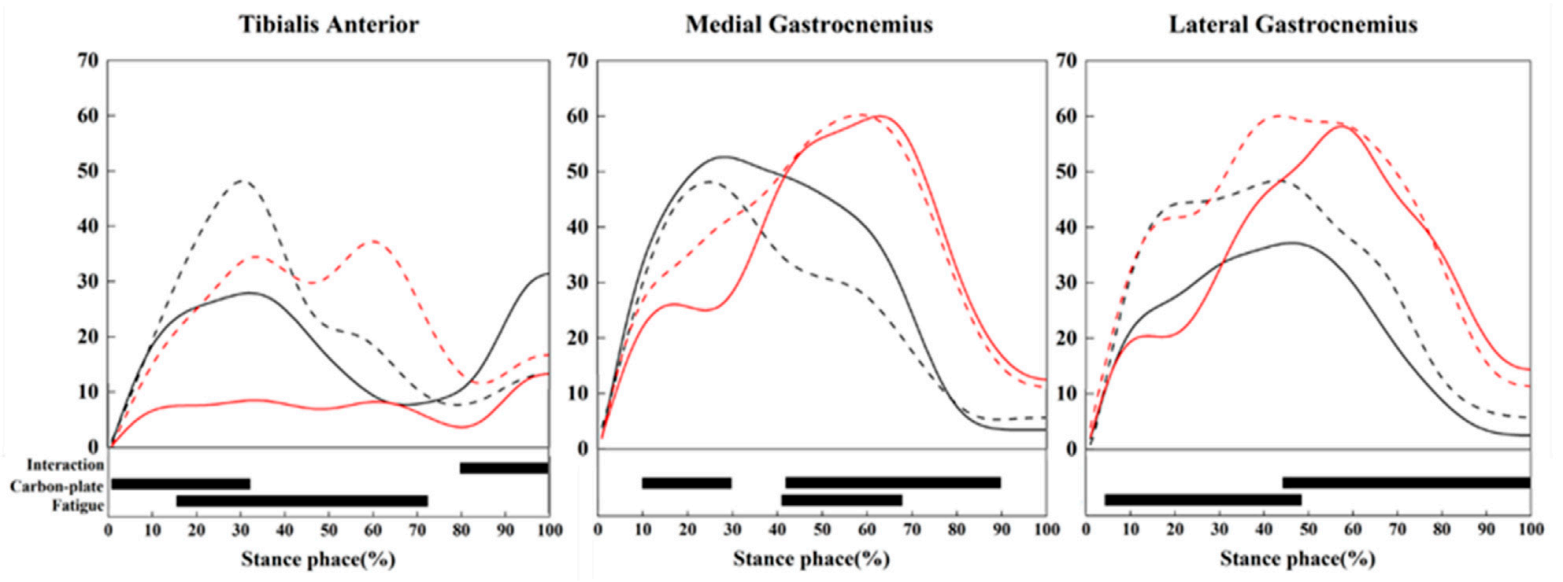
Activation levels of the TA, MG, and LG. Red indicates wearing “Flat”, black indicates wearing “Curve”; solid lines represent pre-fatigue, and dashed lines represent post-fatigue. The significant main effects of the interaction, the shape and the fatigue are highlighted (black horizontal bars at the bottom of the figure) during corresponding time periods.

The results showed that the interaction between “CFP shape” and “fatigue” had significant effect on the ROM of the knee (p = 0.014) and ankle (p = 0.036), the flexion moment of hip (p = 0.025), dorsiflexion moment of ankle (p = 0.05) (Table 2; Figure 4), ankle negative work (p = 0.036), dorsiflexion power of MTP (p = 0.017) and positive work (p = 0.018) (Table 3). It also significantly affected the activation level of the TA during 80%–100% of the stance phase (p < 0.001) (Figure 5). Post-hoc tests revealed that the interaction between CFP shape and fatigue had a significant effect only on the activation of the TA.

### 3.2 Effect of the “CFP shape” or “fatigue”

The significant effects of CFP shape on biomechanical parameters were observed in the contact angle of hip (p = 0.034), knee (p < 0.000), and average flexion moment of hip (p = 0.010) (Table 1; Table 2; Figure 4). Compared to wearing “Flat”, wearing “Curve” resulted in a significant reduction in the contact angle of knee and hip, and average flexion moment of hip in pre- and post-fatigue. The significant effects of fatigue were found in the average flexion moment of hip (p = 0.008) and contact moment of knee (p = 0.018) (Table 1; Table 2; Figure 4). Compared to pre-fatigue, the average flexion moment of hip was significantly reduced in post-fatigue when wearing both types of CPS. There were no significant differences in power and work for the ankle joint and MTP (Table 3).

**TABLE 1:** VALR (BW/s), joints contact angle (°) and contact moment (Nm/kg) of the hip, knee, ankle, and MTP (Mean ± SD).

Index	Flat		Curve		Main effect fatigue	Main effect carbon	Interaction effect
	PRE	POST	PRE	POST	P value	P value	P value
VALR	79.19 ± 24.36	84.46 ± 24.71	75.48 ± 31.36	82.30 ± 17.39	0.150	0.495	0.844
Hip contact angle	26.64 ± 5.97	26.27 ± 8.56	22.08 ± 10.28	21.72 ± 14.37	0.822	*0.034*	0.994
Hip contact moment	0.94 ± 0.67	0.63 ± 0.48	1.26 ± 0.85	1.07 ± 0.98	0.205	0.187	0.710
Knee contact angle	−25.54 ± 5.87	−25.55 ± 6.23	−20.85 ± 5.08	−21.82 ± 8.04	0.618	*0.000*	0.490
Knee contact moment	0.22 ± 0.16	0.15 ± 0.17	0.32 ± 0.19	0.19 ± 0.17	*0.018*	0.253	0.449
Ankle contact angle	−4.00 ± 11.24	4.00 ± 15.00	3.96 ± 5.86	5.05 ± 12.12	0.185	0.098	0.057
Ankle contact moment	−0.07 ± 0.10	−0.03 ± 0.12	0.00 ± 0.10	−0.01 ± 0.07	0.488	0.241	0.062
MTP contact angle	13.76 ± 12.71	11.93 ± 13.85	11.96 ± 9.48	5.58 ± 18.87	0.264	0.119	0.053
MTP contact moment	−0.02 ± 0.05	−0.02 ± 0.04	0.00 ± 0.02	−0.00 ± 0.01	0.259	0.218	0.143

PRE, pre-fatigue, POST, post-fatigue, VALR, vertical average loading rate, SD, standard deviation, MTP, metatarsophalangeal joint, bold italic indicated significant effect of the fatigue, the CFP, shape, or the interaction.

**TABLE 2:** ROM (°) and average moment (Nm/kg) of the hip, knee, ankle, and MTP (Mean ± SD).

Index		Flat		Curve		Main effect fatigue	Main effect carbon	Interaction effect
		PRE	POST	PRE	POST	P value	P value	P value
Hip	ROM	44.00 ± 7.03	43.88 ± 6.63	46.00 ± 12.11	46.50 ± 11.88	0.757	0.181	0.783
	Flexion moment	0.97 ± 0.41	0.70 ± 0.34	0.58 ± 0.31	0.54 ± 0.21	0.008	0.010	0.025
	Extension moment	−1.26 ± 0.45	−1.32 ± 0.40	−1.36 ± 0.43	−1.28 ± 0.47	0.899	0.744	0.164
Knee	ROM	26.05 ± 5.58	24.45 ± 6.67	23.61 ± 7.91	26.94 ± 6.66	0.303	0.976	0.014
	Extension moment	1.22 ± 0.22	1.23 ± 0.18	1.09 ± 0.41	1.14 ± 0.3	0.430	0.089	0.473
	Flexion moment	−0. 28 ± 0.09	−0.30 ± 0.15	−0.36 ± 0.13	−0.35 ± 0.13	0.673	0.084	0.519
Ankle	ROM	32.15 ± 5.07	27.87 ± 6.25	30.40 ± 5.82	30.49 ± 6.05	0.217	0.732	0.036
	Dorsiflexion moment	0.02 ± 0.02	0.04 ± 0.04	0.04 ± 0.03	0.03 ± 0.02	0.897	0.603	0.05
	Plantarflexion moment	−2.16 ± 0.23	−2.07 ± 0.32	−2.02 ± 0.14	−1.97 ± 0.25	0.250	0.062	0.591
MTP	ROM	10.21 ± 2.59	10.72 ± 2.44	10.66 ± 3.32	11.33 ± 4.73	0.522	0.573	0.884
	Dorsiflexion moment	0.01 ± 0.01	0.01 ± 0.01	0.01 ± 0.01	0.00 ± 0.00	0.521	0.435	0.217
	Plantarflexion moment	−0.40 ± 0.13	−0.35 ± 0.18	−0.33 ± 0.06	−0.33 ± 0.12	0.161	0.558	0.152

PRE, pre-fatigue, POST, post-fatigue, SD, standard deviation, MTP, metatarsophalangeal joint, bold italic indicated significant effect of the fatigue, the CFP, shape, or the interaction.

**TABLE 3:** Power (W/kg) and work (J/kg) of the ankle and MTP (Mean ± SD).

Index		Flat		Curve		Main effect fatigue	Main effect carbon	Interaction effect
		PRE	POST	PRE	POST	P value	P value	P value
Ankle	Dorsiflexion power	9.50 ± 2.34	8.30 ± 3.08	8.91 ± 2.36	8.53 ± 2.74	0.249	0.769	0.236
	Plantarflexion power	−9.92 ± 4.33	−7.80 ± 4.81	−6.77 ± 3.24	−6.61 ± 3.12	0.350	0.077	0.066
	Positive work	0.99 ± 0.22	0.83 ± 0.27	0.90 ± 0.19	0.86 ± 0.18	0.124	0.630	0.112
	Negative work	−0.77 ± 0.36	−0.58 ± 0.35	−0.51 ± 0.22	−0.51 ± 0.21	0.295	0.083	0.036
MTP	Dorsiflexion power	0.23 ± 0.19	0.25 ± 0.28	0.22 ± 0.30	0.44 ± 0.59	0.125	0.292	0.017
	Plantarflexion power	−0.65 ± 0.49	−0.52 ± 0.48	−0.35 ± 0.16	−0.36 ± 0.28	0.317	0.171	0.213
	Positive work	0.01 ± 0.01	0.02 ± 0.02	0.00 ± 0.01	0.03 ± 0.04	0.053	0.462	0.018
	Negative work	−0.08 ± 0.07	−0.06 ± 0.07	−0.03 ± 0.03	−0.03 ± 0.01	0.233	0.083	0.226

PRE, pre-fatigue, POST, post-fatigue, SD, standard deviation, MTP, metatarsophalangeal joint.

In addition, at different stages of the stance phase, either CFP shape or fatigue had significant effects on the activation levels of the TA, MG, and LG. From 0% to 30%, the activation level of the TA was higher when wearing “Curve” compared to “Flat”. From 10% to 30%, the activation of the MG was higher when wearing “Curve”, while from 40% to 90%, MG activation was higher when wearing “Flat”. For the LG, activation was higher in “Flat” than in “Curve” during 45%–100% of the stance phase. Regarding the effect of fatigue, from 15% to 42%, TA activation was higher in post-fatigue compared to pre-fatigue. From 5% to 48%, LG activation was significantly higher in post-fatigue than pre-fatigue. Between 40% and 68%, fatigue had a significant effect on MG activation (Figure 5).

## 4 Discussion

This study compared the differences in lower extremity kinematics, kinetics, and muscle activation levels before and after fatigue in 12 male recreationally elite marathon runners who wore two different carbon-plated running shoes. Previous research has demonstrated that these biomechanical indicators are associated with marathon runners’ performance and the risk of running-related injuries. While previous studies have examined the effects of variations in the carbon plate embedded in different running shoes on running performance, the combined effects of carbon plate shape and running fatigue on lower extremity biomechanical characteristics remain unclear. Therefore, the purpose of this study was to investigate the effects of different CFP shapes in running shoes on the lower limb biomechanical characteristics following running-induced fatigue. The main results indicated that, compared to “Flat”, wearing “Curve” resulted in a decrease in the hip and knee contact angles and a reduction in the hip flexion moment. However, there were no significant effects on power and work of joints, which contradicted our hypothesis. In terms of muscle activation, the interaction between CFP and fatigue significantly affected the activation of the TA. Pre-fatigue, the activation level of the TA was higher when wearing “Curve” than when wearing “Flat”, while the opposite was true following running-induced fatigue. This study will examine the impact of different carbon plate shapes in running shoes on lower extremity biomechanical characteristics before and after running fatigue, thereby providing data to support performance enhancement and injury prevention in runners.

The VALR have been retrospectively associated with various running-related overuse injuries. The VALR was selected as an evaluation metric to compare ground impact under varying conditions; however, the results did not indicate significant differences, contradicting our hypothesis (Table 1). In Lieberman’s study, compared to shod running, barefoot running reduced VALR by adjusting the landing pattern (Lieberman et al., 2010). Furthermore, Cheung compared VALR while running on a treadmill with different inclines, both shod and barefoot, and found differences in results, but these were not due to the presence or barefoot, but rather because the landing pattern had changed (An et al., 2015). Compared to footwear properties, changes in VALR may be more strongly influenced by landing patterns. Upon observation, it was found that the landing patterns of the runners in this study did not change under different conditions. The results aligned with previous studies, thus explaining the lack of significant differences in the VALR.

Cigoja et al. (2021) compared the biomechanical data of “Curve” with “Flat” from other experiments (Hoogkamer et al., 2019; Hunter et al., 2019), including stance time, ankle and MTP work, angular velocity, and angles, and found comparable results. However, in our experimental results, there were significant differences in joint angles and moments at the hip and knee joints between the two CFP shapes. Our results showed that, compared to “Flat”, wearing “Curve” significantly reduced the hip contact angle (p = 0.034) and knee contact angle (p < 0.000) (Table 1). In the comparison by Zhou et al. (2021) between high-cushioned bionic shoes and normal shoes, it was found that using bionic shoes caused greater knee and hip flexion than normal shoes. The flexion moment at the hip joint was significantly lower when wearing “Curve” in pre- and post-fatigue than with “Flat” (p = 0.010) (Table 2; Figure 4). Under both CFP conditions, the knee joint contact moment in pre-fatigue was significantly lower than post-fatigue (p = 0.018) (Table 1), while no significant differences were observed in other lower limb joint moments. The research indicates that stiffer shoes can increase hip joint moments (Bergmann et al., 1995), potentially leading to hip joint injuries. Footwear can alter the forces applied by muscles on the lower limb joints and may reduce injury risk by alleviating loads on the joints (Chughtai et al., 2018). The CFP in running shoes is designed to enhance running efficiency by mimicking the principles of levers (Roy and Stefanyshyn, 2006). The significant changes observed in hip and knee joint moments and ground contact angles when wearing “Flat” may result from coupling between the MTP and ankle joint movements with other lower limb joints during the propulsion phase (Allan et al., 2020; Miyazaki et al., 2024), which may explain how different CFP shapes can alter the bending angle of the forefoot and subsequently affect gait parameters.

It has been proposed that muscle activation patterns played an important role in the underlying principles that governed a runner’s preferred movement path (Nigg et al., 2017). Later, Hoitz et al. (2020) indicated that when there were significant kinematic differences due to shoe structures (a minimalist, a conventionally cushioned, and a racing flat shoe), muscle activation strategies changed. Nigg et al. (2021) suggested that the embedded “Curve” in running shoes could be seen as a “lever.” When a runner applies force to the forefoot, the pivot point of the plate shifts forward, causing the center of pressure to move forward as well, thus generating a reaction force at the heel. In our study using SPM to investigate muscle activation (Figure 5), we found that the interaction between CFP shape and fatigue significantly influenced TA activation levels only during 80%–100% of the stance phase. Interestingly, during this phase, the activation level of the TA post-fatigue while wearing the “Flat” condition was higher than that observed pre-fatigue, whereas the activation levels pre- and post-fatigue with the “Curve” condition showed the opposite pattern. The reduction in knee joint contact angle and hip joint flexion moment while wearing the “Curve” condition, as compared to the “Flat” condition, required adjustments in the muscle activation strategy of the TA to stabilize movement. Regarding the influence of CFP shape, the peak activation of the MG and LG muscles occurred later and was greater when wearing “Flat” than when wearing “Curve”. This result may be explained by the lever effect of the curved CFP (Nigg et al., 2021). The incorporation of the curved CFP facilitated increased cushioning space in the midsole, thereby enabling a larger ROM in the MTP and reducing the engagement of the hip, knee, and ankle joints. In contrast, flat CFPs were unable to leverage this effect at the forefoot position, requiring greater activation from the lower limb muscles during the later stance phase. Additionally, the reduced height of the cushioning midsole between the flat CFP and the foot led to higher activation levels in the gastrocnemius, owing to the harder impact of the CFP (Kiesewetter et al., 2022). In a study comparing the effects of barefoot running and wearing CPS on muscle activity, Beck et al. (2020) found that increased longitudinal bending stiffness in shoes did not affect muscle activity during running. Similarly, a study on the impact of shoe hardness on lower limb muscle activity found that soft, medium, and hard midsoles did not influence the activity of the medial quadriceps, biceps femoris, and gastrocnemius (Nigg and Gérin-Lajoie, 2011). This suggests that the differences in lower limb muscle activation levels may have resulted from the influence of CFP on the bending angle of the forefoot.

Interpretations of this study should take the following limitations into account. First, we did not conduct stiffness testing on the running shoes. Although the foam midsoles and CFP were made of the same material but had different shapes, which may affect midsole stiffness (Flores et al., 2019), we did not rule out the potential influence of shoe stiffness on the experimental results. Second, using only VALR as a measure of impact is somewhat limited. Future research could incorporate plantar pressure measurements to assess the impact of CFP on the foot, thereby evaluating injury risk (Willwacher et al., 2022). Lastly, inconsistent with our hypothesis, there was no significant difference observed at the MTP, which may be due to our insufficient precision in studying the MTP. Future studies could improve precision in capturing MTP movement by affixing reflective markers through holes in the shoes (Zhu et al., 2024) to better analyze the effects of carbon plate shape on the MTP.

## 5 Conclusion

Different CFP shapes and running-induced fatigue have a significant impact on lower limb biomechanics. Compared to wearing “Flat,” wearing “Curve” resulted in a significant reduction in the contact angle of the knee and hip, as well as the average flexion moment of the hip in pre- and post-fatigue. Compared to pre-fatigue, the average flexion moment of the hip was significantly reduced post-fatigue when wearing both types of two CPS. The interaction affected the activation level of the TA during 80%–100% of the stance phase. Overall, the curved CFP altered the bending angle of the forefoot, thereby significantly reducing the joint angles and joint moments of the hip and knee. In summary, running shoes incorporating curved carbon plates demonstrate greater potential in enhancing running performance and reducing the risk of running-related injuries. In the future, additional variations of curved carbon plate running shoes could be incorporated, and female recreational elite runners could be included as research participants, thereby enhancing the generalizability of the results.

## Data Availability

The raw data supporting the conclusions of this article will be made available by the authors, without undue reservation.
